# Opioid receptor desensitization: mechanisms and its link to tolerance

**DOI:** 10.3389/fphar.2014.00280

**Published:** 2014-12-18

**Authors:** Stéphane Allouche, Florence Noble, Nicolas Marie

**Affiliations:** ^1^Laboratoire de Signalisation, Électrophysiologie et Imagerie des Lésions D'ischémie-Reperfusion Myocardique, Université de Caen, UPRES EA 4650, IFR 146 ICORECaen, France; ^2^Centre National de la Recherche Scientifique, ERL 3649Paris, France; ^3^Institut National de la Santé et de la Recherche Médicale, UMR-S 1124Paris, France; ^4^Université Paris Descartes, Neuroplasticité et Thérapies des AddictionsParis, France

**Keywords:** opioid receptors, desensitization, tolerance mechanisms, biased signaling, receptor trafficking

## Abstract

Opioid receptors (OR) are part of the class A of G-protein coupled receptors and the target of the opiates, the most powerful analgesic molecules used in clinic. During a protracted use, a tolerance to analgesic effect develops resulting in a reduction of the effectiveness. So understanding mechanisms of tolerance is a great challenge and may help to find new strategies to tackle this side effect. This review will summarize receptor-related mechanisms that could underlie tolerance especially receptor desensitization. We will focus on the latest data obtained on molecular mechanisms involved in opioid receptor desensitization: phosphorylation, receptor uncoupling, internalization, and post-endocytic fate of the receptor.

## Introduction

Opioids are the most potent drugs used for pain relief. However, their therapeutic potential could be limited as a protracted use will lead to tolerance to analgesic effects requiring escalating doses that is associated with side effects such as respiratory depression. A huge work has been devoted to decipher molecular mechanisms of tolerance. It is now well-established that opioid receptors (OR) desensitization and its molecular mechanisms are intimately connected to this phenomenon. Since the beginning of the 1980's when the parallel between tolerance and desensitization has been evoked, many studies came out on the molecular mechanisms underlying OR desensitization. The number of publications related to OR desensitization increased dramatically with the cloning of the opioid receptor 10 years later. In this review, we made an effort to summarize a large amount of these data and point out conflicting results by discussing about the initial conditions (cell models, agonist treatments…). We also integrated the latest developments obtained on the role of receptor trafficking in desensitization and tolerance and the concept of biased agonism.

## Structure and function of opioid receptors

### Different types of opioid receptor

The idea that opiate narcotic analgesics must bind to specific sites or opiate receptors, in the central nervous system and elsewhere, in order to elicit pharmacological responses dates back for half a century. It was based on the finding that there are important structural and steric constraints on most of the actions of opiates. Thus, Beckett and Casy (1954), and Portoghese (1965) postulated the existence of multiple OR based on the relationship between molecular structure of opiate drugs and their analgesic activity. Opioid-binding sites in the central nervous system were demonstrated in mammalian brain tissue in the 1970s by using radioligand-binding assays on isolated brain tissue (Pert and Snyder, 1973; Simon et al., 1973; Terenius, 1973), followed by the characterization of endogenous opioid peptides (Hughes et al., 1975; Cox et al., 1976; Guillemin et al., 1977; Goldstein et al., 1981). The endogenous opioid system, whose involvement in different physiological functions has been recently reviewed (Bodnar, 2014), consists of four distinct neuronal systems that are widely distributed throughout the CNS and peripheral organs. To date, four OR have been cloned, the mu, kappa, delta and nociceptin/orphanin FQ receptor (Evans et al., 1992; Kieffer et al., 1992; Chen et al., 1993a,b; Meng et al., 1993; Thompson et al., 1993; Fukuda et al., 1994; Mollereau et al., 1994). This latter, despite its sequence homology with the first three ones, poorly binds peptide and alkaloid opioid ligands (Mollereau et al., 1994; Reinscheid et al., 1995). So, only data on mu (MOR), delta (DOR), and kappa (KOR) OR will be included in this review. The endogenous opioid peptides are generated from four precursors: proopiomelanocortin, proenkephalin, prodynorphin, and pronociceptin/orphanin FQ (Nakanishi et al., 1979; Kakidani et al., 1982; Noda et al., 1982; Meunier et al., 1995; Reinscheid et al., 1995), each generating biologically active peptides that are released at the synaptic terminals of opioidergic neurons. These peptides exert their physiological actions by interacting with the various classes of OR present on both pre- and post-synaptic membranes of opioid and opioid target neurons (Besse et al., 1990).

Receptor subtypes of mu, delta and kappa OR have been proposed from the pharmacological *in vitro* and *in vivo* studies, but at present there is no molecular evidence to account for a further subclassification. Only one molecular entity for each receptor has been cloned from a given species (Knapp et al., 1995; Dhawan et al., 1996), although functional splice variants of MOR have been discovered (Abbadie et al., 2004; Pasternak et al., 2004; Pan et al., 2005; Pasternak and Pan, 2013). Recent explanations, not mutually exclusives, regarding the diversity of pharmacological responses following activation of a single target, have emerged with the identification of OR heterodimers that appear to have properties different from the monomeric receptors (Fujita et al., 2014; Massotte, 2014; Ong and Cahill, 2014) and the notion of biased agonism (see this review and Violin et al., 2014).

### Structure

Opioid receptors belong to the class A of G protein-coupled receptors (GPCR) which share some common features. They possess seven transmembrane domains linked by three intracellular and three extracellular loops, an extracellular amino-terminus and an intracytoplasmic C-terminus tail. The amino-terminus region has putative glycosylation sites. Whereas O- and N-glycosylation seems to be important for DOR maturation and export to plasma membrane (Petaja-Repo et al., 2000), N-glycosylation of MOR doesn't affect its function (Befort et al., 2001; Rostami et al., 2010). The transmembrane domains are composed of a strong proportion of hydrophobic amino-acids organized in alpha helix and demonstrate the highest sequence homology between the three OR (around 70%) (Mollereau et al., 1994). These domains contain cysteine residues that might be important for ligand binding for MOR (Gioannini et al., 1999) but not for DOR (Ehrlich et al., 1998). The three extracellular loops (most divergent in sequence), including the first two ones linked by a disulfide bond would participate in ligand binding (Metzger and Ferguson, 1995). The three intracellular loops would be more involved in G protein interaction (Metzger and Ferguson, 1995; Georgoussi et al., 1997; Megaritis et al., 2000). The carboxy-terminus tail has a low sequence homology between the three OR. It contains putative phosphorylation sites (Ser, Thr, and Tyr) involved in regulation events after ligand binding and a conserved cysteine residue. This latter could be involved in receptor palmitoylation, a reversible post-translational modification that could regulate DOR surface expression for instance (Petaja-Repo et al., 2006). However, in MOR, mutation of the two Cys residues does not affect palmitoylation (Chen et al., 1998).

In the last few months, an important breakthrough has been made with the crystal structures of MOR (Manglik et al., 2012), DOR (Granier et al., 2012), and KOR (Wu et al., 2012) at high resolution. The results obtained by these studies confirmed some previously discovered important characteristics of OR. Pharmacology of OR has been described with the message/address model: the ligand is composed of two parts, one carrying the activity (agonist or antagonist) at the different subtypes of OR, the “message” and one part, the “address,” conveying selectivity toward a given OR (Portoghese et al., 1990). For the opioid peptides, enkephalins, dynorphins and endorphins, the N-terminal tyrosine residue may be considered as the common message and the C-terminal domain presents the variable address. The deep binding pocket responsible for the “message” recognition is conserved between the different OR subtype, whereas the distal binding site responsible for the “address” recognition is divergent (Metzger and Ferguson, 1995; Granier et al., 2012; Manglik et al., 2012; Filizola and Devi, 2013). For instance, the indole group of naltrindole, carrying the selectivity toward DOR, interacts with the Leu7.35 residue. In the MOR, this amino-acid is replaced by a Trp, preventing naltrindole binding by steric hindrance (Granier et al., 2012; Manglik et al., 2012). Interestingly, MOR crystallized in two-fold symmetrical dimer (Manglik et al., 2012) whereas KOR (Wu et al., 2012) and DOR (Granier et al., 2012) were also shown to adopt anti-parallel arrangements. While those data reinforce the existence of OR dimers (Massotte, 2014), one should keep in mind that the non-physiological conditions (i.e., detergents and modified receptors) used for such crystallographic studies could generate artifactual interactions.

### Signaling and biased agonism

OR are mainly coupled to pertussis toxin-sensitive heterotrimeric G_αi/o_ proteins and to a lesser extent to G_z_ (Law et al., 2000). G_α_ and G_βγ_ dimer activate numerous intracellular effectors. The most studied effector is the adenylyl cyclase (ACase) and investigations on OR coupling demonstrated that stimulation of MOR, DOR, and KOR in cellular models or *ex vivo* inhibited ACase mainly via G_i/o_ proteins (Dhawan et al., 1996; Bian et al., 2012). One of the fastest responses obtained after OR activation is the regulation of certain types of ionic channels such as the inhibition of voltage-dependent Ca^2+^ channels or activation of potassium channels such as GIRK (G protein-coupled inwardly rectifying K^+^ channels) (Law et al., 2000). Activation of K^+^ channels mediates neuronal membrane hyperpolarization and reduces hyperexcitability. The inhibition of voltage-dependent Ca^2+^ channel blocks neurotransmitters release. These two phenomena participate to reduce nociception mediated by OR. OR also activate phospholipase C and mitogen-activated protein (MAP) kinases pathways (Law et al., 2000).

Recently, a new notion has emerged from pharmacological studies of GPCR, called biased agonism or functional selectivity. The binding of different ligands of a single receptor results in distinct conformational changes of receptor; each conformation preferentially interacts with selective partners producing specific signaling cascades (Kenakin, 2011). One could trace back the first data on biased agonism for OR when some authors demonstrated that different ligands for the same OR activate different subsets of Gα_i/o_ proteins (Allouche et al., 1999a). Recently, Morse and colleagues revealed a functional selectivity using a large panel of opioid ligands by the label-free dynamic mass redistribution technology which is based on the detection of refractive index alterations measured by biosensor-coated microplates (Morse et al., 2013); this suggests that opioid ligands are able to promote different conformational changes of OR. Many studies have demonstrated the existence of a biased agonism for OR at different signaling events including desensitization, phosphorylation, endocytosis, trafficking, and *in vivo* effects (see below) (Raehal et al., 2011; Pradhan et al., 2012; Kelly, 2013).

### *In vivo* function

The anatomical localization of OR in the brain and peripheral tissues has been clearly established using autoradiographic methods with selective radiolabeled ligands and detection of OR transcripts using *in situ* hybridization (Mansour et al., 1995; Dhawan et al., 1996). The different OR are widely distributed throughout the central nervous system that explains the large pharmacological responses observed following administration of opioid agonists.

The highest density of MOR is found in the caudate and putamen, where they exhibit a typical patchy distribution in the rat. High levels of MOR are observed in the cortex, thalamus, nucleus accumbens, hippocampus, and amygdala. Moderate levels are found in the periaqueductal gray matter and raphe nuclei, and low concentrations are seen in the hypothalamus, preoptic area, and globus pallidus (Quirion et al., 1983). MOR are also present in the superficial layers of the dorsal horn of the spinal cord (Besse et al., 1990). This large distribution in both spinal and supraspinal structures, as well as at periphery, shows that MOR play an important role in the control of nociception, in good agreement with the pharmacological studies demonstrating that mu selective agonists are potent antinociceptive drugs. Numerous other physiological functions appear to be controlled by MOR. These include reward, respiration, cardiovascular functions, bowel transit, feeding, learning and memory, locomotor activity, thermoregulation, hormone secretion, and immune functions (Dhawan et al., 1996; Kieffer, 1999; Bodnar, 2014).

The distribution of KOR demonstrates some of the most striking species differences among the OR types. In the rat, they represent only approximately 10% of the total number of OR, while in most other species (guinea pig, monkey, and human) they represent at least a third of the opioid binding population (Dhawan et al., 1996). KOR have been found to be widely distributed throughout the forebrain, midbrain, and brainstem. They are implicated in the regulation of several functions, including nociception, diuresis, mood, feeding, and neuroendocrine secretions (Tejeda et al., 2012; Bodnar, 2014).

Compared to MOR and KOR, DOR are more restricted in their distribution and are densest in forebrain regions, well-conserved across mammalian species. Dense binding is observed in the caudate, putamen, cerebral cortex, and amygdala, while they are generally sparse to inexistent in thalamus and hypothalamus. They play a role in different functions: nociception, locomotor activity, gastro-intestinal motility, olfaction, cognitive function, and mood driven behavior (Dhawan et al., 1996; Gaveriaux-Ruff and Kieffer, 2002; Bodnar, 2014).

## Desensitization

Chronic opioid use leads to tolerance, defined as a decrease of the drug response. It's possible to reproduce *in vitro* such phenomenon when cellular models expressing OR are exposed to agonists; in that situation, a decrease of signaling is observed and is designated as OR desensitization. Some reports distinguish the OR desensitization from the cellular tolerance. When rats are chronically exposed to morphine, examination of MOR activity on the outward potassium current shows a reduction compared to naive animals which is not reversible even after 6 h in free-morphine medium; this is cellular tolerance (Levitt and Williams, 2012). In contrast, desensitization may be defined as a reduction of signal transduction from OR after acute activation by agonists that recovers when cells or tissues are placed in agonist-free medium. The first works studying the molecular mechanisms underlying OR desensitization were reported more than 30 years ago (Gahwiler, 1981; Law et al., 1982).

Initially, studying desensitization was made possible by using experimental models endogenously expressing OR such as brain membranes, rabbit cerebellum or cell lines (NG 108-15, SH-SY5Y, SK-N-SH, SK-N-BE…). Since the cloning of the first OR, those models have been superseded by heterologous expression systems (HEK, CHO, COS-7, *Xenopus laevis* oocyte) in which OR are easily expressed in large amount but whose cellular characteristics are far from neurons in which OR are endogenously expressed.

Desensitization of OR is studied on different signaling pathways including ACase inhibition, activation of MAP kinases, inhibition of voltage-gated calcium channels and activation of GIRK channels. Desensitization is sometimes evaluated by measuring the ability of OR to activate G proteins in [^35^S]GTPγS binding experiments after opioid agonists exposure. In absence of modification on the downstream signaling pathway, G protein uncoupling is a good marker for desensitization but can't be applied for G protein-independent pathways (i.e., MAP kinases). The comparison between desensitization studies suffers also from the various experimental conditions used. Cellular model, agonist, agonist concentration, time of exposure, level of OR expression or signaling pathway studied are among the different parameters that could influence OR desensitization as previously reviewed (Connor et al., 2004).

### Definition

As indicated above, desensitization is defined as a progressive reduction of signal transduction that occurs more or less rapidly after OR activation depending on the agonist and the signaling pathway. The rapid desensitization is mainly observed on the regulation of ion channel conductance from sec to several minutes while a sustained desensitization is rather observed on regulation of enzymes (ACase, MAP kinases) after minutes to several tens of minutes. However, in this latter case, other counter-regulatory mechanisms (internalization, traffic of OR) could participate to desensitization making its description complex. Molecular mechanisms turned out to be complicated for several reasons:

– A single OR can activate simultaneously different signaling pathways such ACase, MAP kinases or ion channels and it is possible to observe different levels of desensitization when considering those cellular responses. For instance, we recently showed that remifentanil, a MOR selective agonist, produces a significant desensitization by 60% on the cAMP pathway after 10 min while at the same time desensitization of the MAP kinases ERK1/2 signaling pathway was not significantly affected (Nowoczyn et al., 2013).
– Two types of desensitization, homologous and heterologous, were described. In homologous desensitization, only agonist-activated receptors are desensitized while in heterologous desensitization, both agonist-activated and non-activated receptors sharing the same signaling pathways are inactivated. Those types of desensitization are related to different mechanisms especially in terms of receptor phosphorylation and kinases (Chu et al., 2010). Cross-desensitization between OR and other GPCRs is not systematically investigated and when it is, the level of desensitization between GPCRs using the same signaling pathway can be different (Namir et al., 1997). Recently, Xu et al. showed a cross-desensitization between the dopamine D1 receptors and DOR. This heterologous desensitization characterized by an uncoupling of G proteins from DOR is neither associated with modifications in receptor number nor in their phosphorylation but involves several kinases [cAMP-dependent protein kinase (PKA), MAP kinases/ERK kinase 1 (MEK1) and phosphoinositide-3 kinase (PI3K)] that could phosphorylate signaling proteins (Xu et al., 2013).
– Desensitization results from several regulatory mechanisms of signal transduction and depends on the number of active receptors at the cell surface, the efficiency of OR/G proteins coupling and the post-endocytic traffic. Recently, desensitization of MOR expressed in the neurons from locus coeruleus was demonstrated to result from a decrease of both number of active receptors and the affinity of residual receptors for the agonist (Williams, 2014).

This part will discuss recent data from literature regarding desensitization of the different OR: the impact of the agonist used through the notion of biased agonism, the role of phosphorylation and consequently the kinases involved, the implication of arrestins and OR internalization and their fate after endocytosis. Regarding MOR, a recent review has been published concerning the molecular mechanisms involved in its regulation (Williams et al., 2013).

### Effect of biased agonism on OR desensitization

The first reports describing a differential desensitization of MOR, DOR, and KOR by various agonists came from Reisine's group (Blake et al., 1997a,b; Bot et al., 1997) suggesting that biased agonism could influence desensitization; but at that time this concept was not established yet. Few studies have been designed to evaluate the impact of biased agonism on OR desensitization. They would require determination of the relationship between agonist concentration and the response from a large panel of ligands. More generally, the comparison of the ability of two ligands to promote OR desensitization is realized using the same concentration regardless their intrinsic efficacy.

#### Biased agonism at MOR and desensitization

Functional studies revealed that [D-Ala^2^-MePhe^4^-Gly^5^-ol]enkephalin (DAMGO) induced a stronger desensitization of MOR than morphine in different experimental models and signaling pathways (Yu et al., 1997; Whistler and Von Zastrow, 1998; Koch et al., 2001; Blanchet et al., 2003; Bailey et al., 2009). However, such difference was not reported by others (Liu and Prather, 2001; Borgland et al., 2003; Schulz et al., 2004). In contrast, morphine was demonstrated to promote a stronger MOR desensitization than DAMGO on the increase of intracellular [Ca^2+^] (Chu et al., 2010). In another model, the human neuroblastoma SH-SY5Y, it is possible to observe a huge difference in MOR desensitization produced by morphine and remifentanil on the cAMP pathway but not on the MAP kinases ERK1/2 (Nowoczyn et al., 2013). All those discrepancies could be due to the different level of OR expression, the cellular models and the existence of spare receptors as previously mentioned (Connor et al., 2004).

#### Biased agonism at DOR and desensitization

Evidence for a different DOR regulation by methadone and morphine was also reported; a pretreatment with methadone but not with morphine produced a cross-desensitization with [D-Ala^2^, D-Leu^5^]-enkephalin (DADLE) and morphine (Liu et al., 1999a). Similar data were reported by Bot et al. (1997). In our laboratory, we also showed a differential regulation of human DOR (hDOR) on both the inhibition of ACase and the phosphorylation of ERK1/2 in the SK-N-BE cells. Initially, we suggested that peptidic opioid agonists such as [D-Pen^2^-D-Pen^5^]-enkephalin (DPDPE) and deltorphin I (H-Tyr-D-Ala-Phe-Asp-Val-Val-Gly-NH_2_) induced a stronger and faster desensitization compared to the alkaloid agonist etorphine (Allouche et al., 1999b). However, using other peptidic ([Leu^5^]- and [Met^5^]-enkephalins and UFP-512 ([H-Dmt-Tic-NH-CH(CH_2_-COOH)-Bid])) and non-peptidic (SNC-80 ((+)-4-[(alpha R)-alpha-((2S,5R)-4-allyl-2,5-dimethyl-1-piperazinyl)-3-methoxybenzyl]-N,N-diethyl-benzamide) and ARM-390) ligands we didn't confirm such assumption but our data rather suggest that DOR selective agonists promote profound desensitization compared to non-selective ligands (Marie et al., 2003a; Lecoq et al., 2004; Aguila et al., 2007).

#### Biased agonism at KOR and desensitization

Very few studies examined the regulation of KOR by different agonists. The group of Pei showed that desensitization of KOR-mediated extracellular acidification response was greater upon dynorphin A (1-13) stimulation than U69,593 ((+)-(5α,7α,8β)-N-Methyl-N-[7-(1-pyrrolidinyl)-1-oxaspiro[4.5]dec-8-yl]-benzeneacetamide) and etorphine (Ling et al., 1998). On the cAMP pathway, U50,488 (trans-(±)-3,4-Dichloro-N-methyl-N-[2-(1-pyrrolidinyl)cyclohexyl]benzeneacetamide) and dynorphin A (1-17) produced a greater KOR desensitization than etorphine or levorphanol (Blake et al., 1997b).

With respect to desensitization, all those data support the idea that agonists are able to promote a different regulation of OR as demonstrated for other GPCR such as the histamine H2 receptors (Alonso et al., 2014). Such differential desensitization demonstrated for each OR by different agonists is probably related to the set of different regulatory molecular mechanisms (see above).

### Mechanisms of OR desensitization

#### OR phosphorylation

**Figure 1:**
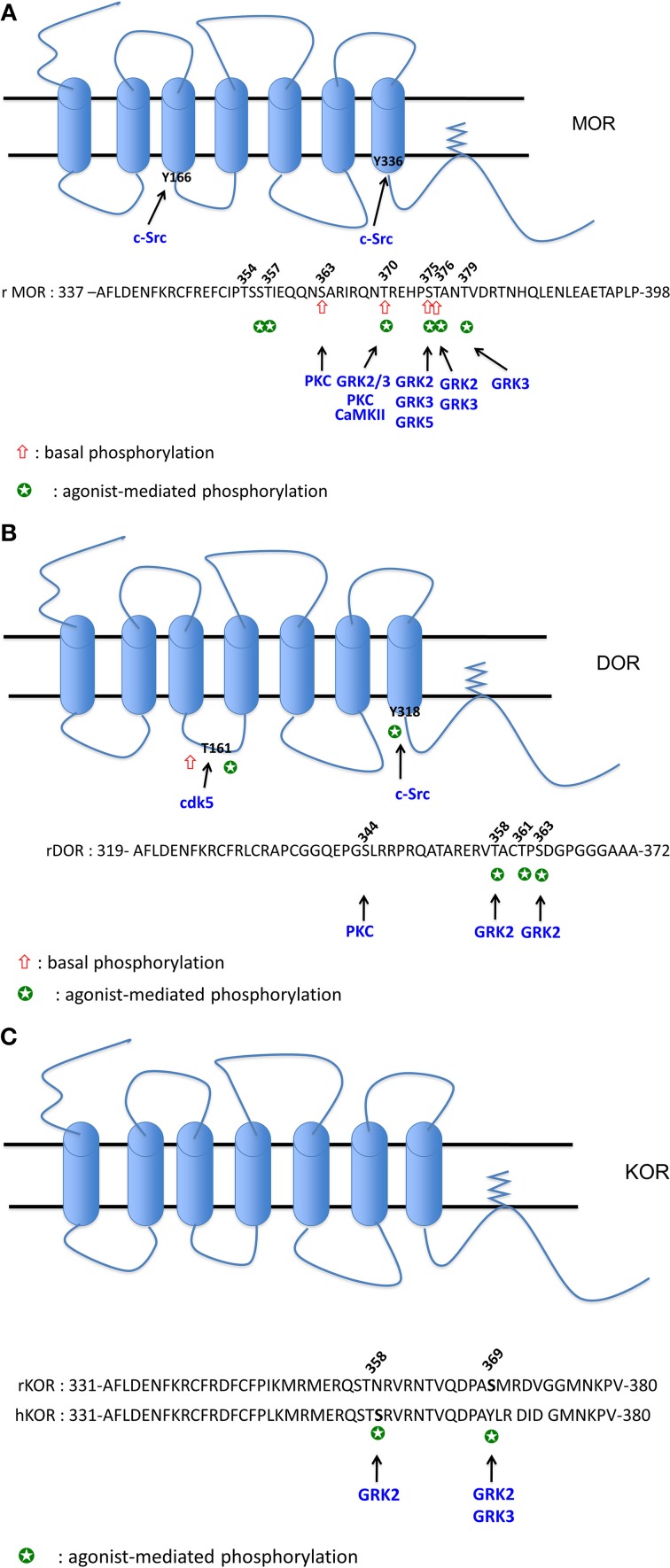
**(A)** Phosphorylation sites of MOR. The cluster 354TSST357: phosphorylation both by DAMGO and morphine (Lau et al., 2011). The S356 (equivalent to Ser358 in human) is phosphorylated by DAMGO (Moulédous et al., 2012), S356 and T357 are phosphorylated both by DAMGO and morphine (Chen et al., 2013). The S363 (equivalent to S365 in human) is phosphorylated in the absence of agonist (El Kouhen et al., 2001; Lau et al., 2011; Moulédous et al., 2012; Chen et al., 2013). PKC was demonstrated to phosphorylate S363 (Chen et al., 2013; Illing et al., 2014). The T370 (equivalent to T372 in human) is phosphorylated in the absence of agonist (Moulédous et al., 2012; Chen et al., 2013). A decrease of phosphorylation level is observed upon DAMGO and 1Dme (a neuropeptide FF analog) exposure (Moulédous et al., 2012). PKC (Illing et al., 2014) and CaMKII (Chen et al., 2013) phophorylate T370. DAMGO, morphine and etonitazene increase phosphorylation at T370 (Doll et al., 2011; Lau et al., 2011). DAMGO-mediated phosphorylation at this residue is ultra-rapid (20 s) (Just et al., 2013) and involves GRK2 and 3 (Doll et al., 2012) but not PKC (Illing et al., 2014). The cluster 375STANT379 displays higher level of phosphorylation upon DAMGO compared to morphine (Lau et al., 2011). S375 or T376 (equivalent to S377 and T378 in human) are phosphorylated upon DAMGO and 1Dme (Moulédous et al., 2012), DAMGO, etonitazene, and morphine (Doll et al., 2011). S375 is considered as the major phosphorylation site as it is rapidly phosphorylated (20 s) upon DAMGO (Just et al., 2013). This agonist-mediated phosphorylation does not implicate PKC (Illing et al., 2014) but rather GRK2 (Chen et al., 2013) or GRK2 and 3 (Doll et al., 2012) upon DAMGO exposure, and GRK5 and to a lesser extent GRK3 upon morphine treatment (Doll et al., 2012). T376 (equivalent to T378 in human) is phosphorylated upon DAMGO and 1Dme (Moulédous et al., 2012), by GRK2 and 3 upon DAMGO exposure but it is considered as a late phosphorylation site (20 min) (Just et al., 2013). T379 is also phosphorylated upon DAMGO exposure after 1 min and required the GRK3 (Just et al., 2013). Y166 (Clayton et al., 2010) and Y336 (Zhang et al., 2009) are phosphorylated by Src. **(B)** Phosphorylation sites of DOR. S344 phosphorylation is mediated by a PKC but is not increased by DPDPE (Xiang et al., 2001). S358 and S363 (Guo et al., 2000; Kouhen et al., 2000) are the two major sites of phosphorylation mediated by GRK2 upon DPDPE exposure. Deltorphin II and morphine are also able to increase phosphorylation at S363 (Navratilova et al., 2005). T361 is phosphorylated by DPDPE but after S358 and S363 phosphorylation (Guo et al., 2000; Kouhen et al., 2000). T161 is phosphorylated by CDK5 in the absence and in the presence of chronic morphine exposure (Xie et al., 2009). Y318 is phosphorylated by Src upon DTLET exposure (Kramer et al., 2000b). **(C)** Phosphorylation sites of KOR. Phosphorylation of S369 (rKOR) is mediated by GRK2 (Mclaughlin et al., 2003) and 3 (Mclaughlin et al., 2004) upon U50488 exposure. In hKOR, S358 is phosphorylated by GRK2 when activated by U50488 (Li et al., 2002).

Numerous studies have been carried out to demonstrate the role of OR phosphorylation in desensitization by using chemical inhibitors of kinases, *in vitro* or *in vivo* knock-out (KO) of kinases using siRNA or transgenic mice, over-expression of dominant negative mutants of kinases, amino acid substitution or truncation on OR. While in some studies the phosphorylation state of OR is clearly determined, in most of them and especially those using kinase inhibitors this major information is lacking. All those data are summarized in Figures 1A–C.

***MOR phosphorylation***. Using metabolic labeling with [^32^P] and different mutants at the C terminal tail, the group of Law demonstrated that rat MOR (rMOR) displays a basal phosphorylation at S363 and T370 and DAMGO increases phosphorylation at T370 and S375 (El Kouhen et al., 2001). Those results were recently confirmed using specific antibodies directed against the phospho-S363, phospho-T370 and phospho-S375 (Doll et al., 2011). As demonstrated for the DOR (see below), agonist-induced MOR phosphorylation is carried out hierarchically with first of all the S375, considered as the major phosphorylation site, followed by T370 (El Kouhen et al., 2001). Morphine was also shown to increase S375 [or S377 for the human MOR (hMOR)] phosphorylation (Nowoczyn et al., 2013) but failed to phosphorylate T370 (Doll et al., 2011). Recently, Just and collaborators showed that MOR is sequentially phosphorylated at S375, T370, T379, and T376 by DAMGO. Interestingly, low concentrations of this opioid agonist rather promote phosphorylation at S375 and T379 while a strong phosphorylation of T370 and S375 is observed at higher concentrations (Just et al., 2013).

Phosphorylation studies using liquid chromatography-mass spectrometry techniques have led to the characterization of two regions at the C terminal tail of the MOR (Lau et al., 2011): the first region (amino acid 349–365) can be mono- or bi-phosphorylated at S363 and in the cluster 354TSST357. While the basal phosphorylation of S363 is not modified by agonist exposure, morphine or DAMGO can increase phosphorylation at the cluster TSST. The second region 375STANT379 is mono- or bi-phosphorylated upon agonist exposure. Rather than qualitative differences, DAMGO and morphine were shown to induce marked quantitatively different phosphorylation increase in MOR. Using a similar experimental approach, two laboratories showed that rMOR and hMOR were phosphorylated in the absence of agonist at S363 and T370 (Moulédous et al., 2012; Chen et al., 2013). Moulédous et al. showed that DAMGO increases hMOR phosphorylation at S356, T370, S375, and T376 (Moulédous et al., 2012) while Chen et al. compared the phosphorylation mediated by DAMGO and morphine; these latter showed that both agonists increase phosphorylation at S356, T357, T370, and S375 (Chen et al., 2013).

Different kinases are involved in MOR phosphorylation. Using siRNA against various forms of the G protein-coupled receptor kinase (GRK) family, DAMGO was demonstrated to phosphorylate T370 and S375 by GRK2 and 3 while morphine increases S375 phosphorylation by GRK5 (Doll et al., 2012). In SH-SY5Y cells, hMOR phosphorylation at S377 (the equivalent of S375 for the rMOR) upon DAMGO exposure does not rely on GRK2 suggesting the implication of another kinases (Moulédous et al., 2012). *In vivo*, using KO mice for either GRK3 or 5, morphine rather promotes MOR phosphorylation at S375 by both kinases while only GRK3 was required for fentanyl-induced MOR phosphorylation (Glück et al., 2014). Using the carboxy-terminal region of MOR fused to glutathione S-transferase and purified kinases, PKC, GRK2, and calmodulin-dependent kinase II (CaMKII) were shown to phosphorylate S363, S375 and T370, respectively (Chen et al., 2013). Various PKC isoforms (PKCα, β II, γ, ε) activated by phorbol 12-myristate 13-acetate (PMA) trigger MOR phosphorylation at S363 and T370 but those kinases are not recruited upon DAMGO stimulation (Doll et al., 2011; Feng et al., 2011); those data indicate the role of PKC in the basal and heterologous phosphorylation of MOR (Illing et al., 2014).

The tyrosine kinase Src was also shown to phosphorylate MOR at Y336, located in the NPXXY motif, after sustained morphine treatment followed by naloxone (Zhang et al., 2009). The Y166, located in the DRY motif of the second intracellular loop of MOR, can be phosphorylated by Src but only upon co-activation with DAMGO and epidermal growth factor (EGF) (Clayton et al., 2010).

In summary, those studies revealed that S375 is the main phosphorylation site of MOR but agonists promote a differential and a multi-phosphorylation of this OR as recently reviewed (Mann et al., 2014).

***DOR phosphorylation***. Pei and colleagues were the first to demonstrate that OR could be phosphorylated upon agonist stimulation (Pei et al., 1995). They showed that DPDPE increases incorporation of [^32^P] in a GRK-dependent manner. As shown for MOR, the group of Law showed that DOR was sequentially phosphorylated at S363, T358, and T361 upon DPDPE exposure (Kouhen et al., 2000). Those results were confirmed by another group who also demonstrated the critical role of GRK2 in DPDPE-induced phosphorylation of these residues (Guo et al., 2000; Marie et al., 2008). Deltorphin II is also able to increase S363 phosphorylation at hDOR but to a greater extent than morphine (Navratilova et al., 2005). PKC can phosphorylate DOR at S344 but is not required for DPDPE-induced DOR phosphorylation (Xiang et al., 2001). In a similar way as MOR, DOR phosphorylation of the Y318, located in the NPXXY motif, occurred upon DTLET ([D-Thr^2^-Leu^5^-Thr^6^]enkephalin) exposure in a Src dependent manner (Kramer et al., 2000a,b). The cyclin-dependent kinase 5 (Cdk5), a proline-directed S/T kinase, was demonstrated to mediate basal and morphine-activated DOR phosphorylation at the T161 located in the second intracellular loop (Xie et al., 2009).

***KOR phosphorylation***. Concerning KOR phosphorylation, the data from literature are very scarce. The group of Chavkin showed that rKOR is phosphorylated *in vivo* at S369 by GRK3 upon U50,488 exposure (Mclaughlin et al., 2004) and *in vitro* by GRK2 (Mclaughlin et al., 2003). Upon global evaluation of the hKOR phosphorylation, Li et al. observed that dynorphin A (1-17) and U50,488 promote the highest phosphorylation, etorphine 50% of the maximum and levorphanol failed to induce [^32^P] incorporation demonstrating that opioid agonists have different potencies to phosphorylate this receptor (Li et al., 2003). It is noteworthy that human and rodent KOR differ substantially in the amino acid composition in the C-terminal region; such difference could explain the absence of rKOR phosphorylation when activated by U50,488 (Li et al., 2002). In hKOR, the S358, substituted by N in the rKOR, is the major phosphorylation site mediated by the GRK2 upon U50,488 exposure.

In summary, the phosphorylation sites for each OR were mapped and showed that activation of a given receptor by different agonists results in a specific pattern involving different kinases (Figures 1A–C). Those data are consistent with the model of barcode established for the β-adrenergic receptor, a prototypic GPCR (Nobles et al., 2011), and could determine the selective interactions between the OR and partners such as arrestins.

#### Uncoupling between G proteins and OR

Any process interfering with the interaction between G proteins and OR can lead to reduction of signal transduction intensity. G protein uncoupling can be evidenced by binding studies on cellular membranes using the radiolabeled non-hydrolyzable GTP analog [^35^S]GTPγS which binds to a G protein activated by the complex receptor-opioid agonist.

In CHO cells over-expressing hDOR, deltorphin II (H-Tyr-D-Ala-Phe-Glu-Val-Val-Gly-NH2) pretreatment induces desensitization after 30 min on the ACase inhibition associated with a G protein uncoupling (Navratilova et al., 2007). In the neuroblastoma×glioma (NG108-15) hybrid cells, morphine pretreatment failed to promote uncoupling of DOR from G proteins while methadone did (Liu et al., 1999b). Conversely, after 5 days of chronic morphine exposure, it is possible to observe a complete uncoupling between MOR and its cognate G proteins (Bohn et al., 2000). However, upon acute exposure (30 min) morphine failed to promote a reduction of [^35^S]GTPγS binding compared to DAMGO indicating a great difference between agonists (Whistler and Von Zastrow, 1998). When expressed in the CHO cell line, the hKOR was demonstrated to undergo a time- and concentration-dependent uncoupling from G proteins but with a moderate impact on the inhibition of ACase (a two-fold increase of the EC_50_ value of the KOR agonist U50488) (Zhu et al., 1998).

#### Relationship between OR phosphorylation and desensitization

In most of these studies, the role of OR phosphorylation in desensitization is indirectly demonstrated by using KO mice or kinases chemical inhibitors; in such situations, we cannot rule out the phosphorylation of other signaling proteins involved in regulatory mechanisms of OR. Mutation of putative phosphorylation sites or truncation of the C terminal tail of OR have been extensively used to delineate the role of phosphorylation in desensitization. All those data are summarized in the Table 1.

**Table 1:** **Role of kinases in OR desensitization/tolerance**.

**OR**	**Main results**	**References**
MOR	GRK2-mediated desensitization after DAMGO exposure	Wang, 2000
	DAMGO mediates desensitization in a GRK2-dependent manner while morphine induced-desensitization in a PKC-dependent fashion	Bailey et al., 2009
	Role of PKCε in morphine- but not etorphine-, fentanyl-, and DAMGO-induced desensitization	Zheng et al., 2011
	Role of GRK2 in homologous and heterologous receptor desensitization	Llorente et al., 2012
	Role of GRK2 in heterologous desensitization between MOR and neuropeptide FF receptor	Moulédous et al., 2012
	No evidence for a role of GRK5 in the development of morphine tolerance	Glück et al., 2014
	Staurosporine and GRK inhibitors do not alter desensitization upon [Met^5^]-enkephalin exposure	Arttamangkul et al., 2012
	Role of PI3Kγ in desensitization and tolerance after chronic morphine treatment	Konig et al., 2010
	Role of JNK2 in tolerance and uncoupling after chronic morphine but not fentanyl treatment	Melief et al., 2010
	Role of Src in ACase superactivation after chronic morphine treatment and naloxone addition	Zhang et al., 2009
DOR	GRK2, PKC and a tyrosine kinase are involved in desensitization of hDOR when activated by etorphine	Marie et al., 2008
	Role of GRK6 in DPDPE-mediated desensitization	Willets and Kelly, 2001
	Role of PKC in DOR desensitization upon sustained activation by DADLE and [Leu^5^]-enkephalin	Yoon et al., 1998; Song and Chueh, 1999
	Role of Src in DPDPE-induced DOR desensitization	Archer-Lahlou et al., 2009; Hong et al., 2009
KOR	Expression of GRK3 or 5 alone is not sufficient to promote desensitization	Appleyard et al., 1999
	Role of GRK3 in development of U50,488 induced tolerance	Mclaughlin et al., 2004

***MOR***. Comparison between two truncated MOR in the C terminal tail in HEK cells over-expressing a GRK2 peptide known to block G_βγ_-mediated recruitment of GRK at the plasma membrane suggest that the amino acids sequence 354TSST357 plays a major role in GRK2-mediated MOR desensitization upon DAMGO exposure (Wang, 2000). In locus coeruleus neurons morphine induced MOR desensitization, measured on K^+^ current, in a PKC-dependent manner while GRK2 was required for DAMGO-induced MOR desensitization (Bailey et al., 2009). Such observations were confirmed by others on Ca^2+^ mobilization; PKC-ε was required for morphine-induced MOR desensitization but not upon etorphine, fentanyl and DAMGO (Zheng et al., 2011). Recently, in locus coeruleus neurons and using chemicals activators (phorbol-12,13-dibutyrate and phorbol-12-myristate-13-acetate) or a muscarinic agonist known to activate PKC, acute or sustained desensitization of MOR induced either by morphine or [Met^5^]-enkephalin were demonstrated to differentially required PKC activity but such effects were not inhibited by the potent PKC inhibitor staurosporine (Arttamangkul et al., 2014). Those data suggest that the involvement of PKC in MOR desensitization would be cell-type specific. In the presence of DAMGO or [Met^5^]-enkephalin, the molecular mechanisms involved in MOR desensitization change during brain development. In the locus coeruleus of young rats, those opioid peptides produce heterologous MOR desensitization with α2 adrenoreceptors in a GRK2-dependent manner but independently of its kinase activity; the high GRK2 expression would sequestrate G_βγ_ and interfere with K^+^ channels activation while in mature rats, homologous MOR desensitization would be due to receptor phosphorylation by this kinase (Llorente et al., 2012). GRK2 was also shown to mediate heterologous desensitization by promoting MOR transphosphorylation upon neuropeptide FF receptor activation (Moulédous et al., 2012). The role of phosphorylation in MOR desensitization has been challenged: using staurosporine as a broad spectrum kinase inhibitor and a GRK2-mutant mice, Arttamangkul et al. showed no modification of [Met^5^]-enkephalin-induced receptor desensitization on K^+^ channels in locus coeruleus neurons (Arttamangkul et al., 2012).

The implication of other kinases than GRK and PKC in MOR desensitization was also investigated. The PI3Kγ was demonstrated to be involved in MOR desensitization on the inhibition of voltage-gated calcium channels induced by chronic morphine treatment (Konig et al., 2010). Using chemical inhibitor and KO mice, c-Jun amino-terminal kinase 2 (JNK2) was demonstrated to play a major role in morphine- but not fentanyl-induced G protein uncoupling (Melief et al., 2010).

Some studies were also conducted to identify the amino acids of MOR involved in desensitization. The T180A substitution abolished MOR desensitization compared to wild type but the phosphorylation state of the receptor was not evaluated (Celver et al., 2004). The S375 was shown to play a major role in MOR desensitization on the cAMP and MAP kinase pathways but only when activated by morphine but not DAMGO (Schulz et al., 2004). Activation of PKC by PMA but not DAMGO pretreatment is able to promote MOR uncoupling from G proteins which is attenuated by the S363A mutation (Feng et al., 2011); this indicates that PKC-mediated phosphorylation of S363 as well as T370 upon substance P receptor activation (Illing et al., 2014) are potentially involved in heterologous desensitization. Using the triple mutant (S363A, T370A, and S375A), Zheng et al. showed that MOR desensitization upon etorphine, fentanyl and DAMGO but not morphine was impaired indicating the different role of amino acids phosphorylation in desensitization (Zheng et al., 2011). As they also demonstrated that PKC mediated morphine-induced MOR desensitization, it can be inferred that PKC would phosphorylate MOR at other sites than S363, T370, and S375. MOR desensitization and phosphorylation at S375 produced by morphine can be modulated by other proteins such as the FK binding protein 12 which would compete with kinase at MOR (Yan et al., 2014).

While all those data indicate that MOR phosphorylation would play a crucial role in desensitization, Qiu and collaborators showed that a truncated mutant of MOR from S363 is able to undergo a similar desensitization to the wild type demonstrating that receptor phosphorylation is not an absolute prerequisite for desensitization (Qiu et al., 2003). However, phosphorylation would rather regulate MOR traffic which could indirectly impact receptor desensitization (see Relationship between OR Internalization and Desensitization).

***DOR***. In SK-N-BE cells, etorphine-induced hDOR desensitization is totally inhibited by using the dominant negative GRK2 mutant K220R but is only reduced when using PKC and tyrosine kinase inhibitors (Marie et al., 2008). In the NG108-15 cell line, rDOR desensitization promoted by a sustained treatment with DPDPE is mediated by GRK6 but not GRK2 as indicated above for hDOR (Willets and Kelly, 2001). The role of PKC in DADLE- and [Leu^5^]-enkephalin-induced DOR desensitization was also demonstrated on the mobilization of Ca^2+^ stores (Yoon et al., 1998; Song and Chueh, 1999). Tyrosine kinases were also suggested to participate in DOR desensitization. Genistein, a broad spectrum tyrosine kinase inhibitor, inhibits hDOR desensitization promoted by DPDPE, deltorphin I, and etorphine (Marie et al., 2008). Hong and collaborators found that DPDPE promotes a tyrosine phosphorylation of DOR which would recruit and activate Src that in turn could phosphorylate and activate GRK2; this latter would then phosphorylates S363 and triggers desensitization (Hong et al., 2009). So, inhibition of Src by PP2 reduces DPDPE-induced DOR phosphorylation of S363 and desensitization on the cAMP pathway but via an indirect mechanism. The role of Src in DOR regulation was also confirmed by the group of Pineyro (Archer-Lahlou et al., 2009).

The major role of DOR phosphorylation at S363 was confirmed using the mutant receptor S363A. While deltorphin II promotes a rapid receptor phosphorylation at this amino acid and desensitization on the cAMP pathway, this latter is totally abolished in the S363A mutant (Navratilova et al., 2007). The T161 of DOR, located in the second intracellular loop and equivalent to the T180 of MOR, also plays a role in DPDPE-induced desensitization; the substitution T161A severely impairs DOR desensitization measured on GIRK channels (Lowe et al., 2002). However, those authors did not evaluate the phosphorylation at this residue. The importance of phosphorylation in DOR desensitization was challenged by the work of Qiu and colleagues who studies those processes using a DOR mutant in which all Ser/Thr residues in the C-terminus region were mutated to Ala (Qiu et al., 2007). They observed that DPDPE-induced desensitization on the inhibition of ACase was significantly delayed but not abolished. This indicates that other mechanisms than phosphorylation could contribute to receptor desensitization.

***KOR***. In the Xenopus oocyte expression system, examination of rKOR regulation on the activation of potassium channels revealed that over-expression of GRK3 or 5 alone did not promote a significant desensitization which requires both GRK and arrestin 3 (Appleyard et al., 1999). This was confirmed when rKOR and GRK2 were co-expressed in CHO cells; pretreatment with a high concentration of U50,488 failed to promote KOR uncoupling from G proteins (Li et al., 2002). Truncation of the C terminal tail of the receptor or the substitution S369A severely impaired U69,593-induced desensitization. These data were further confirmed when wild type and mutant rKOR were expressed in the pituitary adenoma cell line atT-20 cells (Mclaughlin et al., 2003). As indicated above, S358 is the major phosphorylation site for hKOR and the S358N substitution totally abolished U50,488-induced receptor uncoupling from G proteins (Li et al., 2002).

While most of those studies with either indirect or direct proofs indicate the role of OR phosphorylation in desensitization, some of them clearly ruled out such paradigm. This probably indicates that phosphorylation is not a prerequisite for desensitization but would accelerate such process.

#### Role of arrestins in OR regulation

From the canonical model of GPCR regulation by Lefkowitz, arrestins (arrestins 2 and 3 also named β-arrestins 1 and 2, respectively) play a pivotal role in receptor regulation by promoting G protein uncoupling and receptor endocytosis (Pierce et al., 2002). As expected, those proteins were also demonstrated to regulate OR functions. Indeed, over-expression of arrestin 2 induces a selective uncoupling of DOR and KOR and reduces inhibition of ACase (Cheng et al., 1998). However, no significant impact was observed for MOR explaining the lower desensitization rate compared to DOR (Lowe et al., 2002). In recent studies using BRET (Bioluminescence Resonance Energy Transfert) or FRET (Fluorescence Resonance Energy Transfer) techniques, a large panel of opioid ligands were shown to have a different ability to both activate G proteins and recruit arrestins at MOR and DOR (Mcpherson et al., 2010; Molinari et al., 2010; Rivero et al., 2012). For instance, morphine was demonstrated to behave as a partial agonist for DOR and MOR in G protein coupling experiments while almost no interaction with arrestins was detected. This indicates that all opioid ligands do not have the same potency to promote OR desensitization.

***Relationship between arrestins and OR desensitization***. Genetic ablation of arrestin 3 significantly reduces MOR uncoupling from G proteins upon chronic morphine treatment (Bohn et al., 2000). Using dorsal root ganglion neurons from arrestin 3 KO mice, the role of this protein in mediating inhibitory regulation of MOR by JNK on voltage-dependent calcium channels was evidenced (Mittal et al., 2012). This report suggests that arrestin 3 and not arrestin 2 would promote MOR desensitization by interacting with JNK. However, in dorsal root ganglion neurons obtained from arrestin 3 KO mice, acute MOR desensitization elicited by DAMGO or morphine on the inhibition of voltage-gated calcium channels was not significantly different from wild type mice indicating that arrestin 3 has no major role in those conditions (Walwyn et al., 2007). Similarly, in neurons from locus coeruleus no significant role of arrestin 3 was evidenced in acute MOR desensitization upon [Met^5^]-enkephalin exposure on the activation of K^+^ currents (Dang et al., 2009). Yet, concomitant inhibition of arrestin 3 expression (arrestin 3 KO mice) and ERK1/2 activity by PD98059 results in reduction of MOR desensitization indicating that this process involves two independent pathways. In the Xenopus oocyte, over-expression of arrestin alone is not sufficient to increase DOR (Kovoor et al., 1999) or KOR (Appleyard et al., 1999) desensitization while in HEK cells, this over-expression enables morphine-induced MOR desensitization probably by increasing both G protein uncoupling and receptor internalization (Whistler and Von Zastrow, 1998). However, such potentiation could be obtained either when arrestin and a GRK are co-expressed or when the constitutive active arrestin mutant R169E is present. This suggests that OR phosphorylation is a pre-requisite for arrestin action. This conclusion is in good agreement with the data obtained by Johnson et al. on MOR desensitization (Johnson et al., 2006). The translocation of arrestin-2-GFP from cytosol to plasma membrane is only observed upon DAMGO exposure which promotes MOR phosphorylation by GRK2. In contrast, no such translocation could be detected in morphine-treated cells which produce a PKC-dependent MOR desensitization. The use of mouse embryonic fibroblast (MEF) from single or double KO mice for arrestins 2 and 3 revealed that DOR desensitization induced by DPDPE relies predominantly on arrestin 3 expression suggesting a preferential interaction between DOR and this arrestin isoform (Qiu et al., 2007). In the SK-N-BE cells, DOR desensitization is reduced when arrestin 2 expression is inhibited by shRNA only upon DPDPE and deltorphin I exposure but not with etorphine (Aguila et al., 2012).

All those data indicate that different mechanisms are responsible for OR desensitization: some are arrestin-dependent and requires GRK while others are arrestin-independent.

#### OR internalization

The number of active OR at the cell surface is regulated by two processes: endocytosis and export of neosynthesized receptors. Intuitively, when OR internalization is stimulated by agonist exposure, one could expect a reduction in signal transduction. However, the relationship between the number of OR and the cellular response is not linear.

Internalization of OR has been demonstrated in different models with different technical approaches but some discrepancies have been reported. U50,488 and dynorphin A (1-17), but neither etorphine nor levorphanol, promote a time-, and concentration-dependent internalization of hKOR (Li et al., 2003). In several reports, morphine was described as a poor internalizing agonist of MOR in HEK cells (Keith et al., 1998; Whistler and Von Zastrow, 1998; Schulz et al., 2004; Just et al., 2013) but also in enteric neurons (Anselmi et al., 2013) and in brain slice from transgenic mice expressing a FLAG-tagged MOR (Arttamangkul et al., 2008). In few publications, MOR was shown to internalize upon morphine exposure. This was demonstrated for the endogenous MOR in striatal neurons (Haberstock-Debic et al., 2005) and occurred mainly in dendrites (Haberstock-Debic et al., 2003), in the human neuroblastoma cells SH-SY5Y (Nowoczyn et al., 2013) and in double KO MEF for arrestins transfected both with MOR and arrestin 3 (Groer et al., 2011); in those latter publications, morphine-induced receptor internalization was observed for longer time treatment compared to DAMGO. Using a quantitative assay, 30 min morphine exposure promotes half of the MOR internalization induced by DAMGO (Mcpherson et al., 2010). In enteric neurons, morphine promotes a weak internalization of MOR compared to DAMGO as indicated above but chronic morphine exposure results in a significant increase in endocytosis (Patierno et al., 2011).

#### Role of OR phosphorylation in internalization

The role of OR phosphorylation in endocytosis was mainly investigated using OR mutants defective in phosphorylation. The truncated MOR from S363, which is not phosphorylated by DAMGO treatment, was shown to internalize but with a slower rate than the wild type receptor during the first 30 min (Qiu et al., 2003). The S375A mutation strongly impairs DAMGO-driven MOR endocytosis (Schulz et al., 2004). The T370A substitution has no significant effect on DAMGO-induced MOR internalization while it inhibits endocytosis triggered by PKC activation (Illing et al., 2014). This suggests that PKC is able to phosphorylate MOR at T370 and promotes its internalization. Conversely, the role of PKC in internalization was ruled out using activators of this kinase in the locus coeruleus neurons expressing the FLAG-tagged MOR (Arttamangkul et al., 2014). Herkinorin, a MOR agonist, is unable to promote both phosphorylation and internalization indicating that the two processes could be linked (Groer et al., 2007). More than a selective phosphorylation on a specific residue of the carboxy-terminal tail of the receptor, the level of MOR internalization would be correlated to the multi-phosphorylation of T370, S375, T376, and T379 (Just et al., 2013).

As demonstrated for MOR, the phosphorylation-deficient DOR mutant (T358A/T361A/S363G) is able to undergo internalization upon DPDPE activation but to a lesser extent than the wild type (Zhang et al., 2005). However, this DOR mutant cannot internalize anymore when arrestin 3 expression is knocked-down suggesting that the non-phosphorylated DOR can internalize but in an arrestin 3-dependent manner. When the major site of phosphorylation of DOR is mutated (S363A), it is possible to observe a deltorphin I-induced endocytosis (Navratilova et al., 2007); however, it is difficult to assume that this mutation has no impact on internalization since no quantitative evaluation was made. This is in contrast with the study of Bradbury et al. who observed a close correlation between the ability of agonists to phosphorylate the S363 and the degree of DOR internalization (Bradbury et al., 2009).

Concerning the rKOR, the phosphorylation-defective mutant S369E is unable to internalize upon U50,488 exposure demonstrating the role of receptor phosphorylation in endocytosis (Mclaughlin et al., 2003).

While those data indicate that MOR and DOR phosphorylation would favor their endocytosis, KOR phosphorylation would be essential to promote its internalization. Other proteins involved in internalization could also be phosphorylated as demonstrated for the MOR. Activation of phospholipase D2 would enhance MOR endocytosis by the activation of p38 kinase which in turn phosphorylates the Rab5 effector early endosome antigen 1 required for this process (Yang et al., 2010).

#### Role of arrestins in OR internalization

The involvement of arrestins in OR internalization was demonstrated by direct (selective knock-down of arrestin expression) or indirect approaches (visualization of arrestin translocation to plasma membrane) (Table 2).

**Table 2:** **Role of arrestins in OR trafficking**.

**OR**	**Main results**	**References**
MOR	Inhibition of DAMGO-induced MOR internalization by a dominant negative mutant of arrestin 2 in striatal neurons	Haberstock-Debic et al., 2005
	Inhibition of etorphine-induced MOR internalization by a dominant negative mutant of arrestin 2	Zhang et al., 1998
	Morphine promotes MOR internalization by arrestin 3 while upon DAMGO exposure both arrestins 2 and 3 are recruited	Groer et al., 2011
	Morphine induces MOR endocytosis only when GRK2 and arrestin 2 are co-expressed	Zhang et al., 1998
	Herkinorin is unable to promote MOR sequestration	Groer et al., 2007
	MOR is still internalized upon [Met^5^]-enkephalin exposure in arrestin 3 KO mice	Quillinan et al., 2011
	The arrestin 3 reduces recycling of MOR upon chronic morphine but not methadone exposure	Quillinan et al., 2011
	Role of arrestin 2 in MOR recycling upon sustained activation by DAMGO but not morphine	Groer et al., 2007
DOR	DOR endocytosis promoted by DPDPE involves both arrestins 2 and 3. Only arrestin 3 can mediate sequestration of a non-phosphorylated DOR mutant	Zhang et al., 2005
	Arrestin 2 preferentially interacts with DOR to induce its sequestration	Qiu et al., 2007
	Arrestin 2 is involved in DOR internalization upon etorphine but not DPDPE or deltorphin I exposure	Aguila et al., 2012
	Arrestin 3 targets DOR to lysosome when activated by SNC-80 but not DPDPE	Audet et al., 2012
KOR	Inhibition of U50,488-induced KOR internalization by a dominant negative mutant of arrestin 2	Li et al., 1999

DAMGO-induced MOR internalization in striatal neurons is impaired by over-expression of a dominant negative mutant of arrestin 2 corresponding to the last 100 amino acids (arrestin 2 319–418) (Haberstock-Debic et al., 2005). Etorphine also induces an arrestin-dependent MOR internalization as shown by the reduction of receptor endocytosis when the dominant negative mutant V53D of arrestin is over-expressed (Zhang et al., 1998). While DAMGO triggers MOR internalization by recruiting either arrestin 2 or 3, morphine selectively interacts with arrestin 3 which is recruited at the plasma membrane to promote MOR internalization (Groer et al., 2011). In HEK cells, morphine is a poor inducer of MOR internalization. Whereas over-expression of arrestin 2 alone has not significant impact, over-expression of GRK2 greatly enhances receptor sequestration; such GRK2-mediated MOR internalization is potentiated when both kinase and arrestin 2 are both co- and over-expressed (Zhang et al., 1998). The lack of MOR internalization upon activation with herkinorin would be due to the absence of interaction between receptor and arrestin 3 (Groer et al., 2007). The constitutive MOR internalization is also arrestin 3-dependent (Walwyn et al., 2007). Whereas those reports indicate the crucial role of arrestins in MOR endocytosis, this was recently challenged by Quillinan et al. who still observed a MOR internalization upon [Met^5^]-enkephalin exposure in arrestin 3 KO mice (Quillinan et al., 2011). In a recent work, the group of von Zastrow showed that after being recruited by the phosphorylated MOR, arrestin 3 acts as a scaffold, promoting ubiquitination of two lysyl residues in the first intracellular loop by the ubiquitin ligase Smurf2 (Henry et al., 2012). Epsin 1, through its ubiquitin-interacting motifs, recognizes the ubiquitinated MOR contained in the clathrin-coated pits and triggers scission of the vesicle from the cell surface. Those data revealed new inter-relations between MOR phosphorylation and ubiquitination with internalization.

DPDPE also enables arrestin-mediated endocytosis of DOR as shown by the partial reduction of internalization when arrestins 2 or 3 are selectively inhibited (Zhang et al., 2005). The triple DOR mutant T358A/T361A/S363G is still able to internalize but only when arrestin 3 is expressed. This could explain the plasma membrane translocation of arrestin 3-GFP observed in the study of Navratilova and colleagues with the S363A DOR mutant (Navratilova et al., 2007). DOR endocytosis is severely impaired in MEFs obtained from single KO mice for arrestin 2 indicating a preferential interaction between those two proteins (Qiu et al., 2007). It is noteworthy that even when expression of both arrestins 2 and 3 expression is inhibited, a weak proportion of DOR is able to internalize. This is in good agreement with data obtained by Aguila and collaborators who showed that inhibition of arrestin 2 expression reduces etorphine-induced hDOR endocytosis but not upon DPDPE or deltorphin I exposure (Aguila et al., 2012).

As demonstrated for MOR and DOR, KOR also undergoes an arrestin-dependent sequestration when activated by U50,488 as shown by the reduction of internalization when the dominant negative mutant arrestin 2 319–418 is over-expressed (Li et al., 1999).

Together, those data indicate that arrestins are key partners of OR internalization but under specific conditions or agonist exposure, other arrestin-independent mechanisms could occur.

#### Relationship between OR internalization and desensitization

Arttamangkul and collaborators studied desensitization on potassium currents and internalization in neurons from locus coeruleus of transgenic mice expressing a FLAG-tagged MOR (Arttamangkul et al., 2008). Three kinds of ligands can be identified: those which promote both desensitization and internalization ([Met^5^]-enkephalin, etorphine, and methadone), those which induce a desensitization without internalization (morphine and oxymorphone) and oxycodone which promote neither desensitization nor internalization. This reveals the absence of any strong association between internalization and desensitization.

In the Xenopus oocyte expression system, it is possible to observe an acute desensitization of DOR on potassium channels (Kir3) elicited by DPDPE without significant internalization measured by surface biotinylation (Celver et al., 2013). When DOR internalization is significantly inhibited by over-expression of the dominant negative mutant of dynamin (K44E), the desensitization promoted by sustained exposure to DPDPE is not altered (Qiu et al., 2007). This is in good agreement with the observation of Marie et al. who showed that hypertonic sucrose solution totally blocks hDOR endocytosis without any impact on DPDPE- and deltorphin I-induced desensitization (Marie et al., 2003b). Likewise, UFP-512 promotes a strong DOR endocytosis after 15 min exposure without significant desensitization on the cAMP pathway (Aguila et al., 2007). However, upon etorphine exposure a partial reduction of hDOR desensitization is measured when internalization is inhibited.

In contrast, the abolition of rKOR internalization by the S369A substitution also inhibits receptor desensitization on potassium currents (Mclaughlin et al., 2003).

Those data demonstrate that desensitization and internalization are usually two independent processes although in some situations a close relationship could be evidenced. Those apparent discrepancies may be related to the different behavior of MOR and DOR in terms of trafficking (see below). For MOR, internalization would rather promotes recycling and resensitization; when blocking endocytosis, desensitization would be increased. In contrast, DOR are preferentially targeted to degradation, and inhibition of endocytosis would reduce their desensitization; however, this assumption assumes that the receptor at the plasma membrane is not uncoupled from G proteins and it's not always the case.

#### OR trafficking

Once internalized, the OR can follow different routes: sequestration into endosomes, recycling back to the cell surface or targeting to degradation.

The group of Von Zastrow was the first to identify a protein, named GASP for GPCR associated sorting protein, which could actively target DOR to lysosome (Whistler et al., 2002). This protein selectively interacts with the C terminal region of DOR, not MOR, that could explain that under certain circumstances, DOR is degraded while MOR is recycled (Tsao and Von Zastrow, 2000; Whistler et al., 2002). The same group also identified a motif localized at the C terminal region of MOR that enables an active recycling (Tanowitz and Von Zastrow, 2003). This sequence is lacking in DOR but the chimeric DOR containing the last 17 amino acids of MOR recycles after DADLE activation in contrast to wild type. Arrestin 3, dynamin and GRK2 also participate to MOR resensitization on the activation of potassium channels in neurons from the locus coeruleus of mice treated during 6 days with morphine (Dang et al., 2011). This could suggest that those proteins would be involved in MOR trafficking after its internalization and that internalization itself contributes to resensitization (Dang and Christie, 2012). Using neurons obtained from the locus coeruleus of transgenic mice expressing a FLAG-tagged MOR, chronic morphine but not methadone during 6 days was shown to inhibit resensitization and recycling after an acute [Met^5^]-enkephalin exposure (Quillinan et al., 2011). Such weak resensitization and recycling return to the level observed in naive mice when arrestin 3 was knocked-down indicating that this protein would also play a pivotal role in MOR trafficking. Arrestin 2 could regulate post-endocytic sorting of MOR upon DAMGO exposure but not morphine by enabling receptor ubiquitination, as described for different GPCRs (Marchese and Trejo, 2013), but also dephosphorylation on the S375 (Groer et al., 2011). The first hypothesis is unlikely since the sorting of the MOR either toward recycling or lysosomal degradation does not rely on receptor ubiquitination (Hislop et al., 2011). The recycling process involves protein kinases as shown by staurosporine, which increases recycling and resensitization after [Met^5^]-enkephalin exposure (Arttamangkul et al., 2012). Resensitization of MOR after [Met^5^]-enkephalin- or morphine-induced acute desensitization but not cellular tolerance involves dephosphorylation mediated by protein phosphatases sensitive to calyculin A but not okadaic acid (Levitt and Williams, 2012). Similarly, Doll and colleagues showed that the rapid MOR dephosphorylation at S375 involves the protein phosphatase 1γ which increases the recycling of receptors contained in endosomes to cell surface (Doll et al., 2012). The role of receptor dephosphorylation was also demonstrated for both recycling and resensitization of DOR after etorphine treatment (Hasbi et al., 2000).

As indicated above, DOR was initially described as a receptor sorted to lysosomal degradation (Tsao and Von Zastrow, 2000). However, etorphine, [Leu^5^]- and [Met^5^]-enkephalins rather promote a recycling of hDOR while DPDPE, Deltorphin I or SNC-80 induce a degradation and a down-regulation (Marie et al., 2003b; Lecoq et al., 2004). This indicates that the differential sorting of DOR either to recycling or degradation pathway depends on the agonist used and refers to the notion of biased agonism. Audet and collaborators found that DOR activated by SNC-80 strongly interacts with arrestin 3 (Audet et al., 2012). Consequently, the receptor is mainly targeted to lysosome while upon DPDPE exposure, interactions between DOR and arrestin 3 are loose allowing receptor recycling. The ability of DOR to recycle also depends on the duration of agonist exposure. For instance, after 30 min of etorphine treatment, DOR recycles while after 4 h this process is severely impaired (Hasbi et al., 2000). Zhang and collaborators showed different mechanisms to explain the differential sorting of DOR (Zhang et al., 2008): when the receptor is phosphorylated by GRK2 and internalized via arrestins it can recycle whereas in a non-phosphorylated form DOR undergoes an arrestin-independent sequestration which is followed by a degradation. As described for MOR, kinases can be involved in OR sorting. Src was shown to inhibit DOR recycling upon DPDPE treatment that would favor desensitization on the cAMP pathway (Archer-Lahlou et al., 2009). Recently, the endothelin converting enzyme-2, localized in endosomes, was shown to modulate recycling of DOR by degrading opioid peptides such as deltorphin II or the opioid peptide bovine adrenal medulla 22 (BAM22), a cleavage product of proenkephalin (Gupta et al., 2014). When this enzyme is inhibited, DOR recycling decreases and consequently, the desensitization increases. It is noteworthy that this enzyme is ineffective when DOR is activated by the endogenous peptide [Met^5^]-enkephalin and has no role on receptor internalization.

### Molecular mechanisms involved in OR desensitization: a unified mechanism?

**Figure 2:**
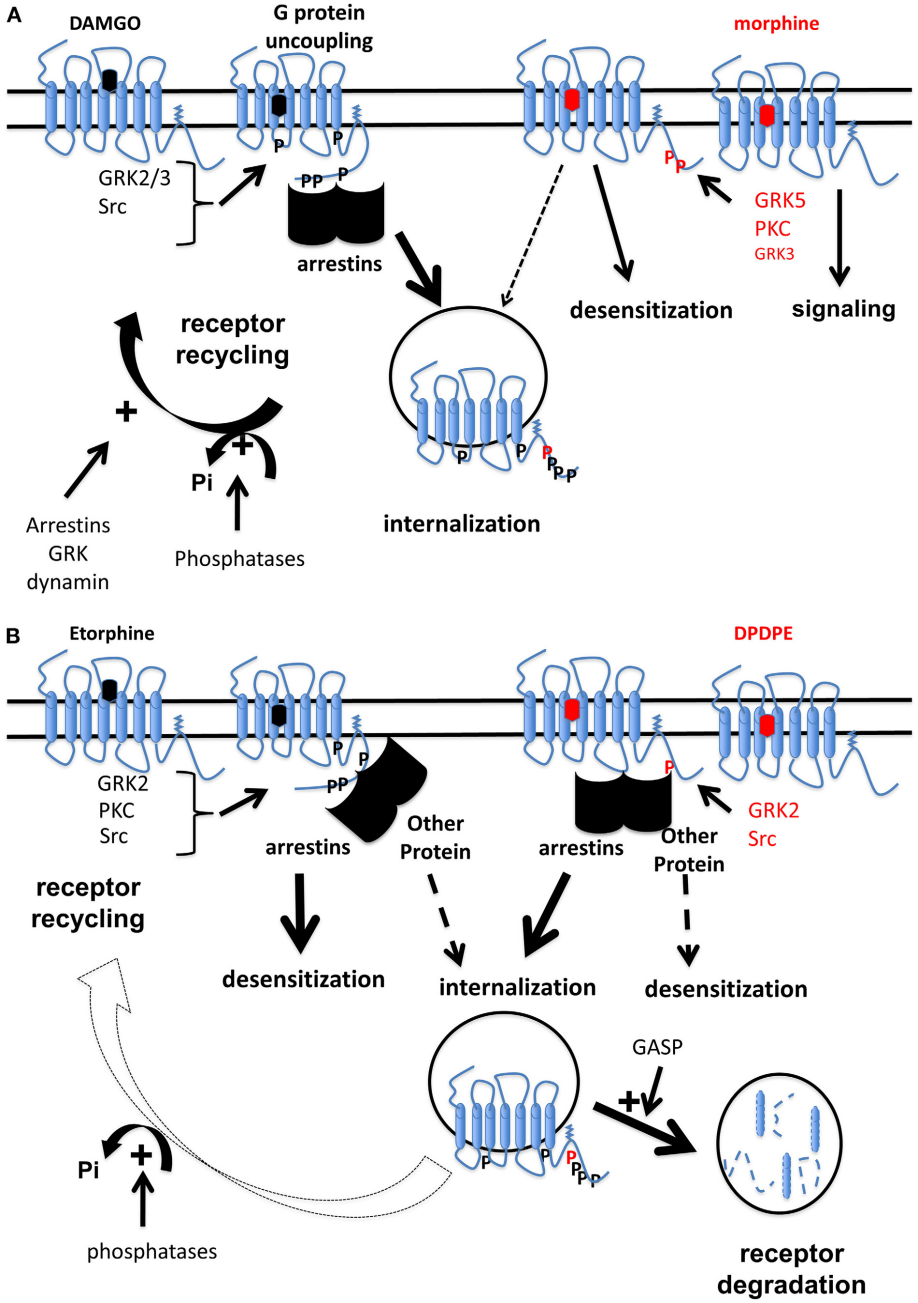
**Schematic illustration of mechanisms involved in opioid receptor desensitization by biased agonists. (A)** MOR are differentially phosphorylated by different kinases upon either DAMGO or morphine exposure (Doll et al., 2011). This results in binding of arrestins to MOR upon DAMGO while this interaction is weakly detectable for morphine when a GRK is over-expressed (Groer et al., 2007). In such conditions, acute DAMGO exposure promotes G protein uncoupling from MOR while morphine does not (Whistler and Von Zastrow, 1998). However, MOR phosphorylation at S375 induced by morphine is able to promote desensitization but not internalization (Schulz et al., 2004). Some reports rather suggest that under morphine exposure, MOR is not desensitized and this continuous signaling promotes tolerance (Finn and Whistler, 2001). Even if it's now well-admitted that morphine is able to promote MOR internalization (Haberstock-Debic et al., 2005; Nowoczyn et al., 2013), DAMGO induces a stronger internalization compared to morphine (Whistler and Von Zastrow, 1998; Schulz et al., 2004). MOR is dephosphorylated by phosphatase proteins (Doll et al., 2012) then undergoes an active recycling (Tanowitz and Von Zastrow, 2003). Other proteins such as arrestins, dynamin, or GRK could participate MOR trafficking (Dang et al., 2011). In contrast, as morphine is a poor inducer of MOR internalization, receptor is maintained in a phosphorylation state at S375 for longer time compared to DAMGO. **(B)** Different kinases are involved in the regulation of hDOR (Marie et al., 2008): GRK2 plays a major role in receptor phosphorylation on S363 upon DPDPE and etorphine while other kinases are also implicated. Etorphine-induced desensitization requires arrestins but not receptor internalization. In contrast, an arrestin is involved in hDOR internalization but not desensitization upon DPDPE (Aguila et al., 2012). Once sequestrated by etorphine, hDOR is dephosphorylated and recycled back to the cell surface (Hasbi et al., 2000; Marie et al., 2003b) while upon DPDPE exposure, the receptor is mainly targeted to lysosomes for degradation (Marie et al., 2003b) probably by a mechanism involving GASP (Whistler et al., 2002).

The vast majority of studies on OR desensitization demonstrated that phosphorylation of OR constitutes a rapid and ubiquitous regulatory mechanisms. However, as illustrated for MOR, quantitative (Lau et al., 2011) or qualitative (Just et al., 2013) differences in MOR phosphorylation were reported upon DAMGO and morphine exposure and those differences in multi-site phosphorylation would result in differential interactions with partners. Conversely, some studies using phosphorylation-deficient receptor challenged this paradigm (Qiu et al., 2003). OR phosphorylation should rather be viewed as a potentiating mechanism that would increase binding of regulatory proteins such as arrestins to the receptor. Mechanisms of desensitization share common features (phosphorylation, accessory proteins involvement such as arrestin, importance of endocytosis and receptor trafficking) and will dependent not only on agonist (biased agonism) but also on time exposure, cell system and receptor. All those mechanisms are depicted in Figures 2A,B.

## Opioid tolerance

### Definition

Drug tolerance is the body's ability to protect itself against the presence of a drug. It is generally observed after protracted exposure but also after acute treatment (acute tolerance) and it is not observed for all the pharmacological effects. For opioids, tolerance to analgesia has been primarily studied as it is the main issue in clinical practice. In rodent, the ability of opioid to promote analgesia to different type of stimuli could be measured using numerous behavioral paradigms including hot-plate test and tail-flick for thermal nociception (Barrot, 2012). Different parameters could modulate tolerance such as the opioid agonist used (Enquist et al., 2012), duration of treatment (Soignier et al., 2004), doses (Huidobro et al., 1976) and even the pharmacological effect observed (Mohammed et al., 2013). So, it is now established that tolerance to respiratory depression is lower than the tolerance to analgesia (Mohammed et al., 2013) and might explain fatal overdoses (White and Irvine, 1999).

### Opioid receptor-related mechanisms of tolerance

Mechanisms of opioid tolerance are complex and multifaceted. We will focus on the mechanisms directly related to receptor regulation such as down-regulation, G protein uncoupling, desensitization, and internalization. Indeed, other mechanisms contribute to tolerance such as activation of anti-opioid systems (NPFF, NMDA) (Ueda and Ueda, 2009) but they are beyond the scope of this review.

#### Down-regulation

Down-regulation is the reduction of receptor number that may result from receptor internalization followed by their degradation, or decrease in receptor synthesis. So, one could hypothesize that it would contribute to tolerance by diminishing the quantity of available receptor. *In vivo*, chronic treatment with opioids promotes decrease (down-regulation), no change or increase (up-regulation) of OR (Bernstein and Welch, 1998; Stafford et al., 2001; Fabian et al., 2002). When downregulation is observed, tolerance might be measured (Gomes et al., 2002) however in some cases tolerance occurs without receptor downregulation (Polastron et al., 1994). These data suggest that downregulation is not mandatory for tolerance.

#### Desensitization

Desensitization and tolerance are very similar in their definition as they both include the notion of a reduced response after prolonged treatment. So, it is tempting to speculate that desensitization and its mechanisms would occur in tolerant animals. In chronic morphine-treated animals desensitization of OR was measured on ACase (Noble and Cox, 1996) and associated with tolerance to analgesic effects (Polastron et al., 1994). In cellular model, receptor uncoupling from G proteins was demonstrated to participate in desensitization (see above). Such uncoupling was also evidenced *in vivo* after chronic opioid agonist exposure. In knock-in mice expressing DOR-eGFP, a challenge with SNC-80 but not ARM-390 induces a tolerance to analgesic response in a model of inflammatory pain with a concomitant G protein uncoupling in both brain and spinal cord homogenates (Pradhan et al., 2009). Acute and chronic treatment with morphine or fentanyl promotes a similar regulation of MOR. In parallel with analgesic tolerance, the ability of MOR to enhance [^35^S]GTPγS binding was reduced compared to naive animals (Bohn et al., 2000; Melief et al., 2010). When arrestin 3 was knocked-out, morphine tolerance and MOR uncoupling from G proteins was reduced in chronic treated animals (Bohn et al., 2000). Interestingly, this KO did not affect tolerance induced by 5 days treatment with fentanyl, oxycodone or methadone (Raehal et al., 2011).

#### Phosphorylation

Anti-nociceptive tolerance induced by morphine, meperidine, and fentanyl was shown to be reduced by PKC inhibitors while DAMGO-induced tolerance and MOR desensitization was shown to rely on GRK (Hull et al., 2010). Whereas *in vitro* experiments showed that S375 is phosphorylated by GRK5 upon morphine exposure (Doll et al., 2012) and S375 phosphorylation plays a major role in MOR desensitization (Schulz et al., 2004), S375A knock-in mice still present anti-nociceptive tolerance upon acute and chronic exposure to morphine (Grecksch et al., 2011). This could indicate that MOR desensitization and tolerance are two unrelated mechanisms. Recently, the role of GRK in morphine tolerance was also questioned: while morphine predominantly promotes S375 phosphorylation by GRK5, chronic morphine treatment induced similar tolerance in wild type and in GRK5 KO mice while dependence was altered (Glück et al., 2014). Similar results were obtained in GRK3 KO mice, when morphine tolerance to analgesia was unchanged whereas tolerance to high efficacy agonists, such as fentanyl or U50,488, was reduced (Terman et al., 2004; Zhang et al., 2013). Rather than inducing desensitization, a chronic morphine treatment could promote a compensatory increase in intracellular cAMP level (also named cAMP overshoot or ACase superactivation) (Avidor-Reiss et al., 1995) and is believed to play a direct role in tolerance (Duman et al., 1988; Javed et al., 2004). In this situation, Src kinase can be recruited at the lipid raft-located MOR and phosphorylates the Y336 leading to ACase superactivation (Zhang et al., 2009). While the mechanism is still unclear, it could implicate Ras/Raf-1 which change the MOR, a GPCR, into a receptor tyrosine kinase like-complex (Zhang et al., 2013).

#### Endocytosis

Accumulating evidences suggest that OR endocytosis decrease opioid tolerance but by mechanisms not fully understood. The first hypothesis has been built by Whistler's group on the inability of morphine to promote MOR internalization despite its capacity to induce strong tolerance. In this case, during morphine treatment, morphine/MOR complexes would accumulate at the plasma membrane and recruit signaling pathways involved in tolerance such as ACase superactivation and NMDA receptor regulation (Finn and Whistler, 2001; He et al., 2002, 2009). In line with this hypothesis, a knock-in mice, expressing a MOR chimera where the C-terminus tail was replaced by the C-terminus tail of DOR, demonstrated less tolerance after chronic morphine treatment (Kim et al., 2008), correlated to a decrease of tolerance biomarkers (He et al., 2009). One explanation of this result is the termination of signal transduction because the DOR C-terminus tail will target the chimeric MOR to lysosomes (Finn and Whistler, 2001). Such results were confirmed when comparing other opioid agonist, buprenorphine and etonitazene. Indeed, buprenorphine, like morphine induces tolerance to analgesia without promoting MOR endocytosis, whereas etonitazene promotes less tolerance and has the ability to promote MOR internalization (Grecksch et al., 2006). Interestingly, coadministration of morphine with subactive doses of internalizing opioids, DAMGO or methadone, enables morphine-induced internalization of MOR and blocks tolerance development (He and Whistler, 2005). An alternative hypothesis was proposed by Koch and collaborators. They proposed that morphine promotes an accumulation of desensitized MOR at the plasma membrane that would result in an increase in apparent desensitization by inhibiting resensitization and would promote tolerance (Koch et al., 2001, 2005; Schulz et al., 2004). However, they found that in knock-in mice expressing MOR mutant S375A substitution, proposed by these authors to be the primary site of morphine-induced phosphorylation of MOR responsible for desensitization (Schulz et al., 2004), morphine tolerance was not affected (Grecksch et al., 2011). The RAVE (relative activity vs. endocytosis) concept proposed by Whistler et al. (1999) cannot be extended to DOR. In DOR-eGFP knock-in mice, the internalizing agonist, SNC-80 promotes acute tolerance to analgesia correlated with strong internalization whereas ARM-390 a non-internalizing agonist did not induce acute tolerance (Pradhan et al., 2009, 2010). When SNC-80 and ARM-390 are chronically administrated, tolerance to analgesia develops and is dependent on endocytosis with SNC80 but not for ARM-390. Interestingly, no tolerance for locomotor effects or anxiolysis appears in ARM-390-treated animals underlying the fact that biased agonist could be used at the behavioral level. All those data support the role of internalization and mainly recycling in reducing tolerance by allowing a sufficient quantity of functional receptors at the cell surface to produce the biological response. However, some opioid agonists such as herkinorin can promote a long lasting anti-nociception without internalization due to the absence of arrestin 3 recruitment (Lamb et al., 2012).

## Conclusions

All the data presented in this review demonstrated that mechanisms of OR regulation are consistent with the model proposed by Lefkowitz (Pierce et al., 2002): agonist activation, receptor phosphorylation, arrestin binding, G protein uncoupling, desensitization, endocytosis followed by targeting to lysosomes or recycling. More interestingly, they also showed that many variations around this model exist depending on the initial conditions, revealing the complexity of OR regulation now translated to the concept of biased agonism. It's an exciting challenge to gain insight this complexity because it will offer a great opportunity to design new drugs that will be able to target a particular pharmacological effect with limited side effects.

### Conflict of interest statement

The authors declare that the research was conducted in the absence of any commercial or financial relationships that could be construed as a potential conflict of interest.
